# Effect of Temperature, pH and Plasmids on In Vitro Biofilm Formation in Escherichia coli

**Published:** 2018

**Authors:** A. Mathlouthi, E. Pennacchietti, D. De Biase

**Affiliations:** Department of Medico-Surgical Sciences and Biotechnologies, Sapienza University of Rome, Laboratory affiliated to the Istituto Pasteur Italia – Fondazione Cenci Bolognetti, Corso della Repubblica 79, 04100 Latina, Italy; Université de Carthage, Faculté des Sciences de Bizerte, Département des Sciences de la Vie, Laboratoire de Biosurveillance de l’Environnement (LR01/ES14), Unité d’Ecotoxicologie, Route de Tunis, 7021 Zarzouna, Tunisie

**Keywords:** Escherichia coli, biofilm, growth conditions, transcriptional regulators, plasmids

## Abstract

Acid resistance (AR) in *Escherichia coli *is an important trait that protects this microorganism from the deleterious effect of low-pH environments. Reports on biofilm formation in *E. coli *K12 showed that the genes participating in AR were differentially expressed. Herein, we investigated the relationship between AR genes, in particular those coding for specific transcriptional regulators, and their biofilm-forming ability at the phenotypic level. The latter was measured in 96-well plates by staining the bacteria attached to the well, following 24-hour growth under static conditions, with crystal violet. The growth conditions were as follows: Luria Bertani (LB) medium at neutral and acidic pH, at 37°C or 25°C. We observed that the three major transcriptional regulators of the AR genes (*gadX*, *gadE*, *gadW*) only marginally affected biofilm formation in *E. coli*. However, a striking and novel finding was the different abilities of all the tested *E. coli *strains to form a biofilm depending on the temperature and pH of the medium: LB, pH 7.4, strongly supported biofilm formation at 25°C, with biofilm being hardly detectable at 37°C. On the contrary, LB, pH 5.5, best supported biofilm formation at 37°C. Moreover, we observed that when *E. coli *carried a plasmid, the presence of the plasmid itself affected the ability to develop a biofilm, typically by increasing its formation. This phenomenon varies from plasmid to plasmid, depends on growth conditions, and, to the best of our knowledge, remains largely uninvestigated.

## INTRODUCTION

In the last two decades, several reports have greatly contributed to our current understanding of the molecular mechanisms that underlie the acid tolerance response (ATR) and acid resistance (AR) in many neutralophilic bacteria. The literature on this topic has recently been reviewed [1].

Concerning AR, this is defined as the astonishing ability of bacteria in the stationary phase of growth to withstand exposure to extreme acid stress (pH ≤ 2.5) for at least 2 hours (such as the one encountered in the gastric compartment) and recover their growth after a return to neutral pH [2]. In this regard, AR is considered to be a key factor during colonization of a host and the infectious process carried out by the gram-negative bacterium *Escherichia coli*, as well as by other bacteria, including pathogenic ones [3-5]. Four AR systems (AR1-4) have been identified in *E. coli*, the most potent of them being AR2, which relies only on the availability of amino acid *L*-glutamate in the minimal salt medium in which the acid challenge is carried out [3, 6, 7]. In this system, amino acid *L*-glutamate is the substrate of the cytosolic enzyme glutamate decarboxylase (two isoforms, GadA and GadB, are expressed in *E. coli*); *L*-glutamate is imported from the medium by the inner membrane antiporter GadC, which couples the import of *L*-glutamate with export of γ-aminobutyrate (GABA), the decarboxylation product. In fact, during the decarboxylation, the α-carboxylic group of *L*-glutamate is released as carbon dioxide (CO_2_) and is replaced with a proton irreversibly incorporated in the GABA molecule. Therefore, the system works by con suming proton intracellularly (through GadA/B activity) and by exporting positive charges through GadC [1, 6].

The regulation of the AR2 system in *E. coli *is extremely complex: it involves several global regulators, such as RpoS (the sigma factor of RNA polymerase of the stationary phase, which positively affects expression of the system) and H-NS (histone-like nucleoid structuring protein, which represses the relevant genes), small RNAs, and several specific transcriptional regulators, such as GadE, GadX and GadW [3, 6]. These specific regulators are encoded by the relevant genes located in the AFI (Acid Fitness Island), the *E. coli *genome region that carries 14 genes involved in the AR at various levels, including the gene coding for GadA [6]. The coordinated transcriptional control of expression of the AFI and AR2 genes (including *gadB *and *gadC*, which are not in the AFI), as well as the involvement of the global and specific transcriptional regulators, was shown in several transcriptional studies, mostly using microarrays [3]. As expected, some studies showed that *gadBC *and the AFI genes were upregulated under all those conditions, which are compatible with the timely activation of AR, such as inorganic and organic acid stress, respiratory stress/anaerobiosis (typical of the gut environment), whereas downregulation was observed under alkaline stress and in an *rpoS *mutant. Notably, in a temporal study of biofilm formation, *gadB*, *gadC, *and the AFI genes were found to be downregulated and the same trend was observed in a study of a protein involved in AR, YmgB [8].

It is well known that biofilm formation is a very complex process which is affected by many factors, such as the strain under investigation and the nature of the surface on which the biofilm develops. In this report, we used the reference laboratory strain *E. coli *K12 MG1655 and its Δ*gadE*, Δ*gadX*, and Δ*gadW *isogenic derivatives to perform a comparative phenotypic study focusing on the effect of these mutations on the ability of *E. coli *MG1655 to form a biofilm at acidic *vs *neutral pH and under temperatures that closely resemble those of the host (37°C) and non-host/ambient (25°C) environment. In addition, we assessed the effect of empty plasmids, i.e. the ones not carrying a gene *in trans*, on biofilm formation and concluded that, when using a plasmid, caution is waranted regarding the plasmid-specific effect on biofilm formation, depending on the experimental conditions under analysis.

## EXPERIMENTAL PROCEDURES

**Materials**

The ingredients for bacterial growth were from Difco. Crystal violet was from Merck. Acetone, absolute ethanol and polystyrene 96-well plates (untreated) were from VWR. Ampicillin was from Roche Applied Science. Kanamycin was from Fluka, and chloramphenicol was from Sigma-Aldrich.

**Table 1:** Bacterial strains and plasmids used in this study

Bacterial strains	Relevant genotype/information
MG1655	F^-^ λ^–^ rph^-1^
MG1655/pBBR	F^-^ λ^–^ rph^-1^ carrying plasmid pBBR1MCS
MG1655/pBS	F^-^ λ^–^ rph^-1^ carrying plasmid pBS
MG1655∆gadE	MG1655 gadE::Kan^R^
MG1655∆gadX	MG1655 gadX::Kan^R^
MG1655∆gadW	MG1655 gadW::Kan^R^
Plasmids	
pBBR1MCS	Expression plasmid (4707 bp): lac, T3 and T7 promoters, CAT/Cam^R^
pBS	(pBluescriptSK) multicopy phagemid vector; ColE1 replicon, lacZα bla

**Bacterial strains, plasmids and growth conditions**

The bacterial strains and plasmids used in this work are listed in *Table 1*. *E. coli *K12 MG1655 and Δ*gadE*, Δ*gadX*, Δ*gadW *isogenic derivatives ([9] and referenced therein) were grown at 37°C or 25°C in one of the following media: LB (Luria Bertani) broth, pH 7.4; LB-MES, pH 5.5 (LB buffered with 100 mM of 2-(N-morpholino)ethanesulfonic acid, MES, at pH 5.5). When required, the ampicillin, kanamycin, and chloramphenicol antibiotics were added at concentrations of 100, 25, and 34 μg/ml, respectively.

**Conditions for biofilm formation**

The experiments were performed in triplicates, starting from independent bacterial colonies picked from a freshly streaked plate from a bacterial stock at -80°C. Each bacterial culture was prepared by transferring a single colony into 2 ml of LB pH 7.4 and allowing the bacteria to grow overnight (16–18 hours) at 37°C under orbital shaking (120 rpm). On the following day, each culture was diluted 1 : 10 into a fresh LB medium and the optical density (OD) at 600 nm was measured. Each culture was then brought to the same OD_600_ = 2.0 and diluted 1 : 100 in independent wells by transferring 2 μl of each culture into 198 μl of either LB, pH 7.4 or LB-MES, pH 5.5. The starting OD (time 0) was checked using a Tecan Sunrise microplate reader at 595 nm. The plates were then transferred to thermostatic static incubators at 25°C and 37°C, respectively. The external wells in each plate contained sterile water or LB to avoid evaporation, and some wells contained only the growth medium (bk), which was read at time 0 and 24 h. Following growth under static conditions for 24 hours, the final OD_595_ (time 24 h) was read and planktonic bacteria were removed. Each well was rinsed with sterile water three times, and then 200 μl of 0.1% crystal violet was added and allowed to stain the biofilm for 15 min. After removal of the crystal violet and three subsequent washes with sterile water to remove the excess of stain, the stained biofilm was solubilized by adding 200 μl of an acetone:ethanol (20:80, v/v) solution. 125 μl/200 μl were transferred from each well in a clean 96-wells plate. Readings were again performed at 595 nm using a microplate reader.

**Analysis of biofilm formation**

The readings obtained after staining with crystal violet were subtracted from those of the wells containing only the medium (bk at 24 h); the readings were previously checked to be identical to the readings of the medium at time 0 in order to verify that there was no contamination. The net readings were then analyzed using the Prism 4.0 GraphPad software. The data for the biofilms obtained using the mutant strains *vs *the wild-type strains were analyzed by two-way ANOVA using the Bonferroni test (as available in the GraphPad Prism software suite, version v5.0a). The data were expressed as the means of 3 to 8 independent experiments with standard deviations (SD). Differences were considered statistically significant at *P* < 0.05.

## RESULTS AND DISCUSSION

**Effect of temperature and pH of the medium on biofilm formation**

**Fig. 1:**
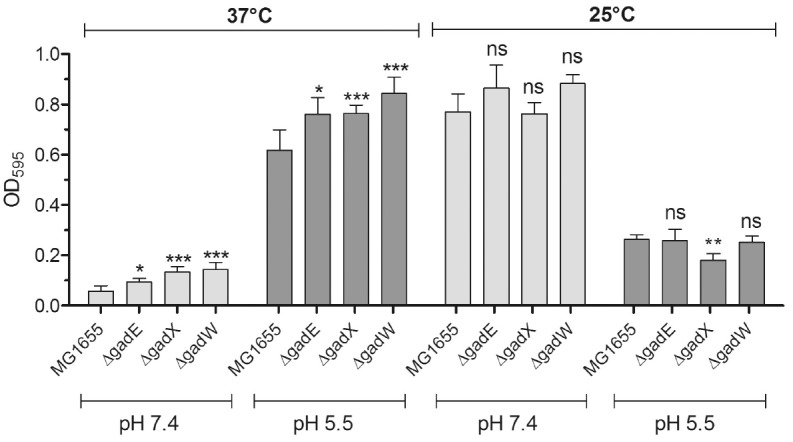
Biofilm formation in *E. coli *MG1655 at different pH values and temperatures. Statistical significance: *, *P *≤ 0.05; **, *P *≤ 0.01; ***, *P *≤ 0.001; n.s., not significant

We analyzed the ability of *E. coli *MG1655 and its Δ*gadE*, Δ*gadX*, and Δ*gadW *isogenic derivatives to form biofilms following growth of bacteria in LB at neutral and acidic pHs at two temperatures, 37°C and 25°C. Strikingly, we noticed that the temperature had a significant effect on biofilm formation for the strain under analysis (*Fig. 1*). In particular, in LB at pH 7.4, biofilm formation was pronounced at 25°C and hardly detectable at 37°C. However, pH of the medium also had an effect, because in LB at pH 5.5 MG1655 formed much more biofilm at 37°C than at 25°C. This phenomenon was only slightly affected by the mutations in the genes coding for the major transcriptional regulators of the AR2 system. This implies that none of these regulators is strongly involved in the transcriptional repression of the genes participating in biofilm formation, at least under our growth conditions. This is in line with the report showing that GadX only marginally affects biofilm biomass in the *E. coli *strain BW25113 [10].

Such striking inversion of the ability to produce biofilms was an unexpected finding. A possible explanation may reside in the pH 5.5, which is more typical of the distal gut. Therefore, the combination of two cues –mildly acidic pH and 37°C –could better approximate the host gut environment, thereby triggering biofilm formation, at least on an inert surface such as polystyrene. The slight increase in biofilm formation observed at 37°C when testing the mutants, regardless of the pH of the medium, could very likely be related to an effect of the regulators on the biofilm structure rather than on the biomass, as noticed by other researchers [10], which may also be related to the observed repression of the AR2 and AFI genes in a temporal study of biofilm formation [11].

**Effect of plasmids on biofilm formation**

**Fig. 2:**
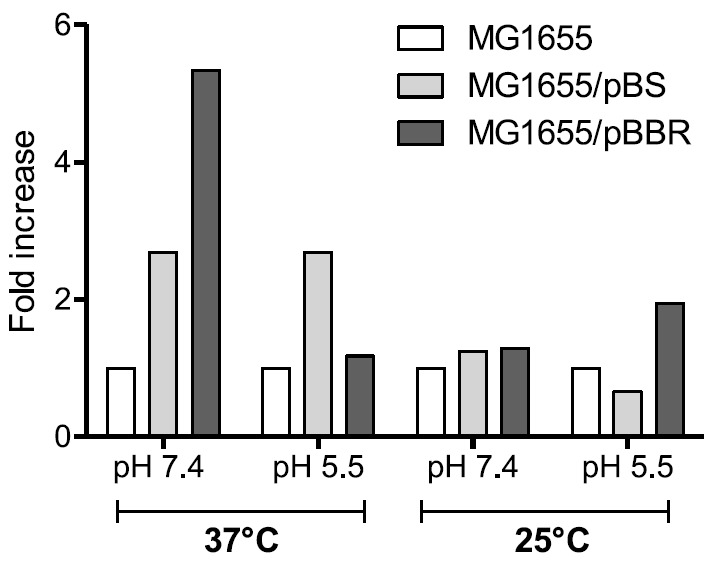
Biofilm formation in *E. coli *MG1655 in the presence of plasmids. The change is reported as fold change increase/decrease with respect to the biofilm formed by the reference strain, i.e. *E. coli*, MG1655 under the indicated condition, which was set to 1.0. The SD of the reported values never exceeded 20% of the indicated value

Another interesting finding derived from the observation of the effect of empty plasmids in bacteria tested for their biofilm-forming ability. In order not to add too many variables, we transformed *E. coli *MG1655 with either a high-copy number plasmid (pBS, in *Table 1*) or a medium-copy number plasmid (pBBR1MCS, in *Table 1*). Biofilm formation was assayed under the same conditions as those shown in *Fig. 1*. The results are shown in *Fig. 2* as fold increase with respect to *E. coli *MG1655 not carrying a plasmid. These data clearly show that both plasmids sometimes exerted a negligible and sometimes a substantial effect on biofilm formation. This phenomenon depended on the medium pH and the temperature and could not be predicted *a priori*.

## CONCLUSIONS

Our results clearly show that pH is an important driving force in dictating the formation of biofilms, to the same extent as temperature. Moreover, care should be taken when interpreting results on *E. coli *strains carrying plasmids that contain a gene complementing a mutation. In fact, we have shown that empty plasmids affect biofilm formation. To the best of our knowledge, this aspect is less investigated than the plasmid transfer within a biofilm [12].

## Abbreviations

LB: Luria Bertani
AR: acid resistance
ATR: acid tolerance response
AFI: acid fitness island
H-NS: histone-like nucleoid structuring protein
MES: 2-(N-morpholino)ethanesulfonic acid
OD: optical density
SD: standard deviation
